# Antidepressant-Like Properties of Fish Oil on Postpartum Depression-Like Rats Model: Involvement of Serotonergic System

**DOI:** 10.3390/brainsci10100733

**Published:** 2020-10-13

**Authors:** Nurul Uyun Abdul Aziz, Samaila Musa Chiroma, Mohamad Aris Mohd Moklas, Mohd Ilham Adenan, Amin Ismail, Mohamad Taufik Hidayat Baharuldin

**Affiliations:** 1 Department of Human Anatomy, Faculty of Medicine and Health Sciences, Universiti Putra Malaysia (UPM), Serdang 43400, Selangor, Malaysia; nurul_uyun@fsskj.upsi.edu.my (N.U.A.A.); musasamailachiroma@yahoo.com (S.M.C.); aris@upm.edu.my (M.A.M.M.); 2 Department of Human Anatomy, Faculty of Basic Medical Science, University of Maiduguri, Maiduguri 600231, Borno State, Nigeria; 3 Atta-ur-Rahman Institute for Natural Product Discovery, Universiti Teknologi Mara (UiTM) Kampus Puncak Alam, Bandar Puncak Alam 42300, Selangor, Malaysia; mohdilham@puncakalam.uitm.edu.my; 4 Department of Nutrition & Dietetics, Faculty of Medicine and Health Sciences, Universiti Putra Malaysia (UPM), Serdang 43400, Selangor, Malaysia; amin@upm.edu.my

**Keywords:** antidepressants, menhaden fish oil, forced swimming test, postpartum depression and serotonergic neurotransmitter

## Abstract

Pathophysiology of postpartum depression (PPD) has been associated with many factors, such as neuroendocrine, neuroinflammation and neurotransmitter changes. Fish oil (FO) improves PPD both in humans and animals. However, little is known with regards to its pharmacology on a PPD-like rat model. Hence, the current study aimed at investigating the effects of FO on a PPD-like rat model. Female rats were induced with PPD-like symptoms and then randomly divided into six groups (*n* = 6) for two experimental protocols. Protocol 1 consisted of PPD-like rats (2 mL distilled water), PPD-like + FO (9 g/kg/d) and PPD-like + Fluoxetine (FLX) (15 mg/kg/d) groups of rats, whereas Protocol 2 consisted of PPD-like rats (2 mL distilled water) + PCPA (p-chlorophenylalanine) 150 mg/kg, PPD-like + FO (9 g/kg/d) + PCPA 150 mg/kg and PPD-like + FLX (15 mg/d) + PCPA 150 mg/kg groups of rats, respectively. All treatments were administered orally for 10 days postpartum, except PCPA, which was given intraperitoneally. Prior to euthanasia, the antidepressant-like effect of the FO was evaluated using the forced swimming test (FST) and open field test (OFT) on day 10 postpartum. Biochemical analysis of serotonin, serotonin metabolite and serotonin turnover from their prefrontal cortex and hippocampus were also measured. The results showed that FO decreased immobility time and increased swimming time significantly, but not climbing time in FST. Further, it also decreased serotonin metabolite and turnover significantly in the hippocampus of the PPD-like rats. In contrast, administration with PCPA reversed all the outcomes. The antidepressant-like effects of FO were found to be similar with that of FLX. Thus, it can be concluded that FO exerts its antidepressant-like effects in PPD-like rats through modulation of serotonergic system.

## 1. Introduction

Postpartum depression (PPD) a familiar problem associated with about 10%–20% of women after child delivery [1]. According to the World Health Organization (WHO), PPD is the most common global mental health problem amongst women of child bearing age, which impacts negatively on infants, mothers, families and society, besides its detrimental effect on the economy [2,3]. There are a number of factors that influence the development of PPD, which include the mental health status of women during pregnancy, levels of ovarian hormones after delivery and the level of social interaction between women with their families and society in general [4]. It has been reported that sudden decrease of ovarian hormones after child delivery and low level of omega-3 fatty acid in postpartum mothers play a contributory role in the development of PPD [5]. Further, alterations of serotonergic system are also strongly associated with the onset of depression [6]. Modulation of serotonin (5HT) and its receptors ameliorates the risk for depression [7]. Hence, the available anti-depressants in the market work by increasing the levels of 5HT at the synaptic junction, which is achieved by inhibiting serotonin transporter (SERT) at synaptic junction. This inhibits the reuptake of 5HT back into the neurons.

Previous studies have shown that, fish oil (FO), a rich source of omega-3 fatty acid, has a therapeutic potential on many diseases, especially those related to mood disorders, as supplementation of FO to both humans [8,9] and animals [10,11] has improved their mental health status. Supplementation of omega-3 fatty acid to humans reduced their risk for PPD [12], while 15 days of FO supplementation to the rat model of PPD exerted antidepressant-like effects [13]. Moreover, in a bulbectomy rat model of depression, ingestion of FO increased the hippocampal level of brain-derived neurotrophic factor (BDNF) and 5HT, which are the two major regulators of neuronal survival and long-term plasticity in the brain [14]. Vines et al. (2012) [15] reported that the antidepressant-like effects of FO are associated with serotonergic system, together with the activation of serotonin receptor, 5HT1A, as the postsynaptic receptor of 5HT1A in hippocampus is involved in the antidepressant-like effects of FO [16]. However, Shukkoor [17] reported that lipid extract of *Channa striatus*, a freshwater fish, has no significant influence on 5HT concentration in both prefrontal cortex and hippocampus of PPD-like rats. Thus, there are no consistent results with regards to the anti-depressant-like effects of FO [5,18,19], while its pharmacology on PPD remains elusive [20].

The exploration of alternative and complementary medicine in combatting the risks of depression is highly suggestive; this can be achieved through the identification and evaluation of the relevant biological markers. Previous studies have shown the antidepressant-like effects of FO rich in omega-3 fatty acid in PPD-like rats [13], although their mechanisms of action are not fully explored. FO supplementation has been shown to exert antidepressant-like effects on PPD-like rats after being administered for 15 days postpartum [13,17]. Therefore, the present study aimed to investigate 10 days of FO supplementation on a PPD-like rat model. The antidepressant-like effects of FO on behavior of the PPD-like rats were evaluated through a forced swimming test (FST) and open field test (OFT). Meanwhile, the concentration of 5HT, its metabolites, 5HIAA and its turnover rate (5HIAA/5HT) in the prefrontal cortex (PFC) and hippocampus were analyzed using enzyme-linked immunosorbent assay (ELISA).

## 2. Materials and Methods

### 2.1. Chemicals

Chemicals used in this study include hormones, estradiol (E8515, Sigma Chemicals, St Louis, MO, USA) and progesterone (P0130, Sigma Chemicals, St Louis, MO, USA) dissolved in 0.1 mL sesame oil (S3547, Sigma Chemicals, St Louis, MO, USA). The specifications of the sesame oil used in this study, as provided by Sigma Chemicals, were as follows: appearance (turbidity): clear; appearance (color): yellow to yellow-green; appearance (form): liquid; identity: conforms; specific gravity: 0.916–0.921; solidification range: 20–25 °C; free fatty acids: (0.02 N NaOH) <2.0 mL; iodine value: 103–116; saponification value: 188–195; unsaponifiable matter: <1.5%; heavy metal: < 0.001%; and cottonseed oil absent: confirmed. Fish oil (F8020, Sigma Chemicals, St Louis, MO, USA) and fluoxetine (FLX) (F132, Sigma Chemicals, St Louis, MO, USA) were also used; other chemicals used for the study include p-chlorophenylalanine methyl ester (PCPA, Sigma Chemicals, St Louis, MO, USA), an inhibitor of the rate limiting enzyme, tryptophan hydroxylase, which is responsible for 5HT production (Vines et al., 2012). Further, ELISA kits for the measurements of 5HT (ST/5HT, E-EL-0033, Elabscience, Houston, TX, USA) and its metabolites 5-Hydroxyindolacetic acid (5HIAA, E-EL-0075, Elabscience, Houston, TX, USA) were also used.

### 2.2. Experimental Animals

Female albino Wistar rats, aged 60–70 days (180–200 g), were purchased at the beginning of the experiment from Saintik Enterprise Company, Malaysia, and housed at the Animal House, Faculty of Medicine and Health Sciences, Universiti Putra Malaysia, Serdang, Selangor, Malaysia. The Animal Experimental Unit (Animal House) lies on a latitude of 2.978705 and a longitude of 101.717662, while the altitude of Serdang, where the animal house is located, is about 0–500 m above sea level. All rats were housed two per cage under 12 hrs/12 hrs light/dark cycles at 22 ± 1 °C with free access to water and standard rat chow (Gold Coin, Malaysia, Table 1). Prior to the experiment, all the rats were acclimatized for 14 days under laboratory conditions. The experiments were conducted from 8 am to 3 pm every day. All experimental procedures were conducted in accordance with the animal ethics guidelines, which were approved by the Institutional Unit of Animal Care and Use Committee (IACUC), Universiti Putra Malaysia, with the reference number UPM/IACUC/AUP-R097/2015.

### 2.3. Construction of PPD-Like Rat Model and Menhaden FO Supplementation

The induction of PPD in rats involves ovariectomy, a hormone-simulated pregnancy (HSP) regime followed by the withdrawal of ovarian hormones; then, confirmation through maternal nesting behavior, as previously described by Arbabi and Shukkoor [13,17], is conducted. Ovariectomy was carried out by a veterinarian; this lowered the risk of the death of rats in the experiment. Both ovaries were removed under anesthesia, ketamine (80 mg/kg intramuscular (i.m)) and xylazine (10 mg/kg i.m). In the maternal nesting behavior test, a paper towel was placed for each rat in its cage to use as a nesting material. The presence of a circular-shaped nest-like structure made from the paper towel observed in the rat’s cage was an indicator of success for the induction of HSP, while absence of a nest-like structure in the cages indicates failure for the induction of HSP and these rats were excluded from the studies. This study recorded a 95% success rate for the induction of HSP, as confirmed by the nesting behavior exhibited by the rats. The supplementation with FO was based on an established protocol [13]. The dose of the FO (Menhaden fish oil, Sigma F8020) was calculated daily using the formula (body weight (g) × 0.009)/density (g/mL) for dose 9 g/kg. Its composition includes 30% omega-3 fatty acid with 10%–15% eicosapentaenoic acid and 8%–15% docosahexaenoic acid, which has been proven to be safe in rats. FO and FLX were administered orally using oral gavage.

### 2.4. Experimental Procedure

In this study, the experimental procedure consisted of two protocols: the first protocol, evaluating the effect of FO on PPD-like rats, and the second protocol, evaluating the effect of FO on PPD-like rats after the administration of PCPA, a 5HT blocker.

**Figure 1:**
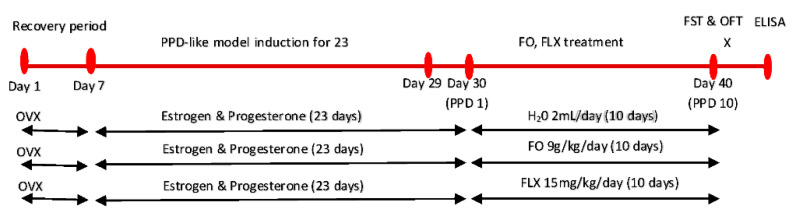
Experimental design for the protocol 1, evaluation of FO on PPD-like rat model. PPD—postpartum depression, FO—fish oil, FLX—fluoxetine, PCPA—p-chlorophenylalanine methyl ester, FST—forced swimming test, OFT—open field test, OVX—ovariectomy, H_2_O—water, X–euthanasia.

**Protocol 1** (Figure 1) was conducted to evaluate the antidepressant-like effects of 10 days of FO supplementation on a PPD-like rat model through FST and OFT, followed by biochemical analysis of serotonin, serotonin metabolite and serotonin turnover. The effects of FO were compared with FLX, which is a common antidepressant readily available in the market. The rats were randomly divided into three groups (*n* = 6).

- PPD-like group (*n* = 6): received distilled water 2 mL/day for 10 days postpartum
- PPD-like +FO group (*n* = 6): received FO 9 g/kg/day for 10 days postpartum
- PPD-like + FLX group (*n* = 6): received FLX 15 mg/kg for 10 days postpartum.

**Figure 2:**
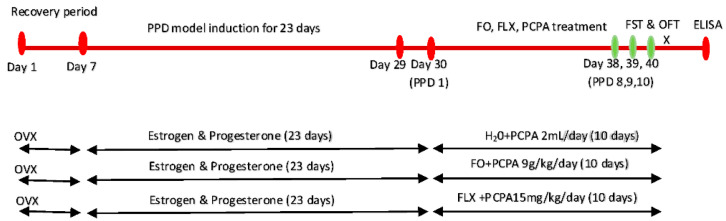
Experimental design for protocol 2, evaluation of FO on PPD rat model after PCPA administration. PPD—postpartum depression, FO—fish oil, FLX—fluoxetine, PCPA—p-chlorophenylalanine methyl ester, FST—forces swim test, OFT—open field test, OVX—ovariectomy, H_2_O—water, X—euthanasia.

**Protocol 2** (Figure 2) was carried out to evaluate the antidepressant-like effects of 10 days of supplementation of FO on a PPD rat model after administration of PCPA on PPD days 8, 9 and 10 through FST and OFT, followed by biochemical analysis of 5HT, serotonin metabolite and 5HT turn over. The effects of FO were compared with FLX. The rats were randomly divided into three groups (*n* = 6).

- PPD-like + PCPA group (*n* = 6); received distilled water 2 mL/day for 10 days + PCPA 150 mg/kg on days 8, 9 and 10 postpartum.
- PPD-like + FO group (*n* = 6); received FO 9 g/kg/day for 10 days + PCPA 150 mg/kg on days 8, 9 and 10 postpartum.
- PPD-like + FLX group (*n* = 6); received FLX 15 mg/kg for 10 days + PCPA 150 mg/kg on days 8, 9 and 10 postpartum.

### 2.5. Behavioral Assessments

#### 2.5.1. Open Field Test

OFT was carried out to evaluate spontaneous locomotor activities of the experimental rats. The locomotor activities of the rats were evaluated by counting the number of small squares (25 squares of equal dimension of 15 cm square each) crossed by the rats at the bottom of an open field arena, a square box measuring 75 × 75 cm length and width, respectively, and 42 cm high. At the beginning of the test, rats were left for 1 h in the animal behavior room for habituation. The lux of the animal testing behavior room ranged from 25 to 100, depending on the number of lights on, but during the testing it was about 25 lux. Then, the rats were placed at the center of the open field arena and were observed for 5 min for their locomotor activities (number of lines crossed) and all changes were recorded using a video camera for later analysis, as described by Chiroma [22]. A rat is considered to have crossed a line when all of its four paws are within one square. The data obtained from the test were rated blindly by two trained independent observers. The average of the data reported by the two observers was used for statistical analysis.

#### 2.5.2. Forced Swimming Test

FST was carried out 1 h after OFT was completed. Three behavioral patterns were assessed through FST; the immobility behavior (a floating-like behavior without any necessary movement, unless for maintaining the rat’s nose above the water level), swimming behavior (an active behavior with horizontal movement of the forelimb or hind limb in a paddling fashion) and climbing behavior (an active behavior with vertical movement of the rats’ front paw that breaks the surface of the water up to the wall). In FST, the time spent on each behavioral pattern by rats after the given treatment was observed in a plastic cylinder (25 cm in diameter and 50 cm height) which contained water 30 cm deep at a temperature of 27 °C. FST was conducted in two sessions; the first session was a pre-test, whereby rats were forced to swim for 15 min to induce the state of helplessness, and the second session was conducted after 24 h, with a 5 min period of swimming. All behavioral changes were recorded using a video camera for subsequent analysis [23,24]. The video obtained was analyzed by two independent raters who were blinded to the rat’s groupings in order to avoid bias. Inter-rater differences were avoided by using the averages of the two raters as the final data for analysis to ascertain the depressive-like behaviors of the rats.

### 2.6. Biochemical Assessments

#### 2.6.1. Tissue Preparations

On day 10 postpartum, the rats were euthanized through decapitation; the whole brain was harvested and placed on a cold plate, followed by rapid isolation of their hippocampus and prefrontal cortex (PFC). Then, the rats were rinsed in cold phosphate buffer saline (PBS) and weighed. The tissues were then homogenized in cold PBS with every 100 mg tissue minced in 1 mL of 1× PBS. The homogenates were then centrifuged for 5 min at 5000× *g* 4 °C. The supernatants were aliquot into small volume and stored at −80 °C for later use.

#### 2.6.2. Assessment of 5HT, 5HIAA and Serotonin Turnover in Hippocampus and PFC

ELISA kits were used to measure the concentration of monoamines neurotransmitter, serotonin (ST/5HT, E-EL-0033, Elabscience, Houston, TX, USA) and its metabolites 5-Hydroxyindolacetic Acid (5HIAA, E-EL-0075, Elabscience, Houston, TX, USA) in brain PFC and hippocampus. Both of the ELISA kits were rat-specific commercialized ELISA kits [25]. The assays were prepared based on the manufacturer’s instruction and the measurements were read using photospectrometer at an optical density 450 nm. Serotonin turnover ratio (5HIAA/5HT) was then calculated using the measurement of 5HT and 5HIAA for both the PFC and hippocampus.

### 2.7. Statistical Analysis

The data obtained were statistically analyzed using SPSS version 23 and GraphPad prism version 6 (ISI, San Diego, CA, USA) software. All the results were expressed as mean ± standard error of mean (SEM). The data were analyzed using one-way analysis of variance (ANOVA) followed by Tukey’s or Dunnett’s post hoc tests, where appropriate, for multiple comparison. A significant difference was accepted when the *p* value was less than 0.05 (*p* < 0.05).

## 3. Results

### 3.1. Effects of 10 Days FO Supplementation on Behaviors of PPD-Like Rats

#### 3.1.1. Ten Days FO Supplementation Improved PPD-Like Rats’ Behaviors in FST

**Figure 3:**
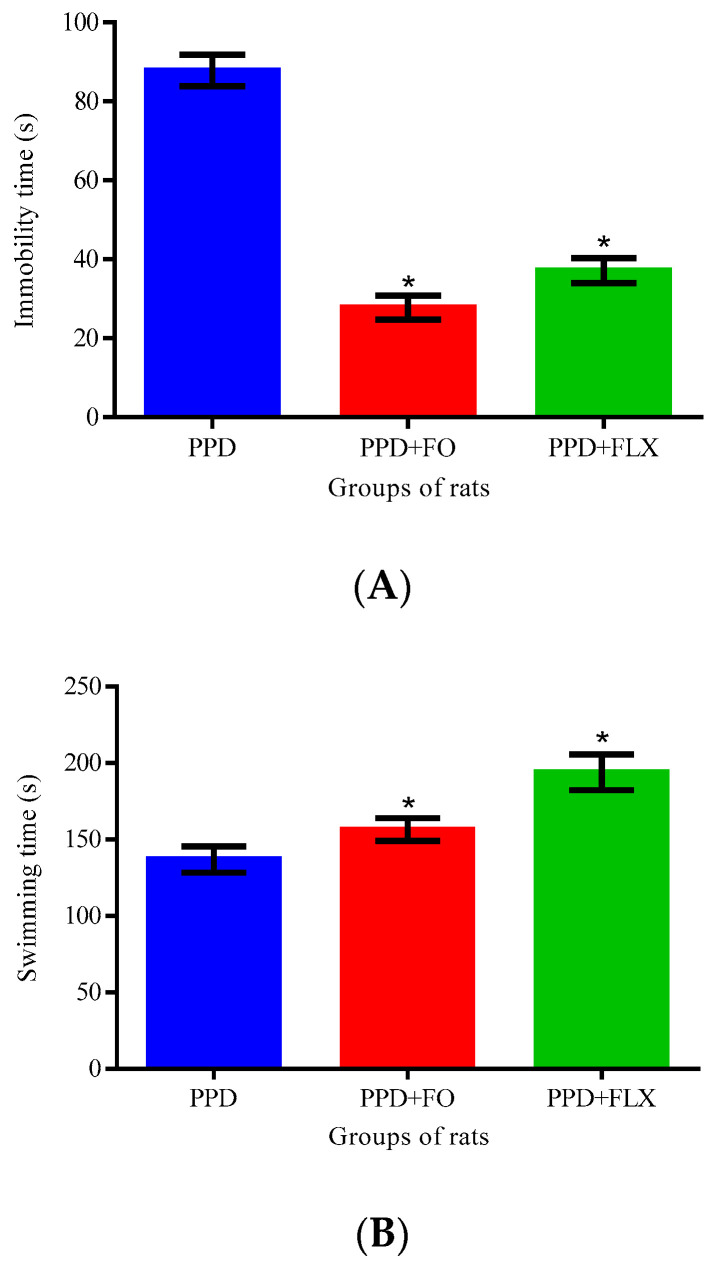

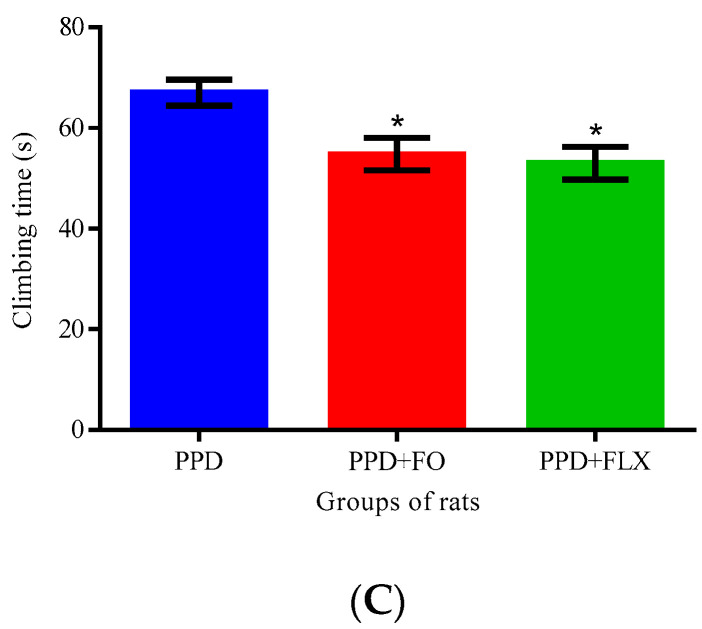
Effect of FO on Forced swimming test in PPD rats. (**A)**—Immobility time. (**B**)—swimming time and (**C**)—climbing time. Data presented as mean ± SEM, *n* = 6, * *p* < 0.05 vs. PPD. (PPD—postpartum depression-like, FO—fish oil, FLX—fluoxetine). Dunnett’s post hoc test was used for the comparisons.

Figure 3 showed the effect of 10 days of FO supplementation on FST in PPD-like rats. One-way ANOVA revealed statistically significant differences in the climbing time, in seconds, of the rats [F (2, 12) = 6.366, *p* = 0.01]. Dunnett’s post hoc test showed a significant decrease of climbing time, in seconds, in the PPD-like + FO (54.8 ± 7.15, *p* = 0.02) and PPD-like + FLX (53 ± 7.20, *p* = 0.01) groups of rats when, compared to the PPD-like model group (67 ± 5.70). Furthermore, one-way ANOVA showed statistically significant differences in immobility time, in seconds, among the rat groups [F (2, 12) = 89.22, *p* = 0.0001]. Dunnett’s post hoc test revealed a significant decrease of immobility time, in seconds, in the PPD-like + FO (27.8 ± 6.72, *p* = 0.0001) and PPD + FLX (37.2 ± 7.19, *p* = 0.0001) groups of rats, when compared to the PPD-like model group of rats (87.8 ± 8.84). Finally, one-way ANOVA showed statistically significant differences in the swimming time, in seconds, among the rat groups [F (2, 12) = 47.24, *p* = 0.0001]. Dunnett’s post hoc revealed a significant increase of swimming time, in seconds, in the PPD-like + FO (156.6 ± 7.53, *p* = 0.01) and PPD-like + FLX (194 ± 11.64, *p* = 0.0001) groups of rats, when compared to the PPD-like model group (137 ± 8.60).

#### 3.1.2. Three Days Administration of PCPA Blocked the Effect of 10 Days Administered FO and FLX on FST Behavior in PPD-Like Rats

**Figure 4:**
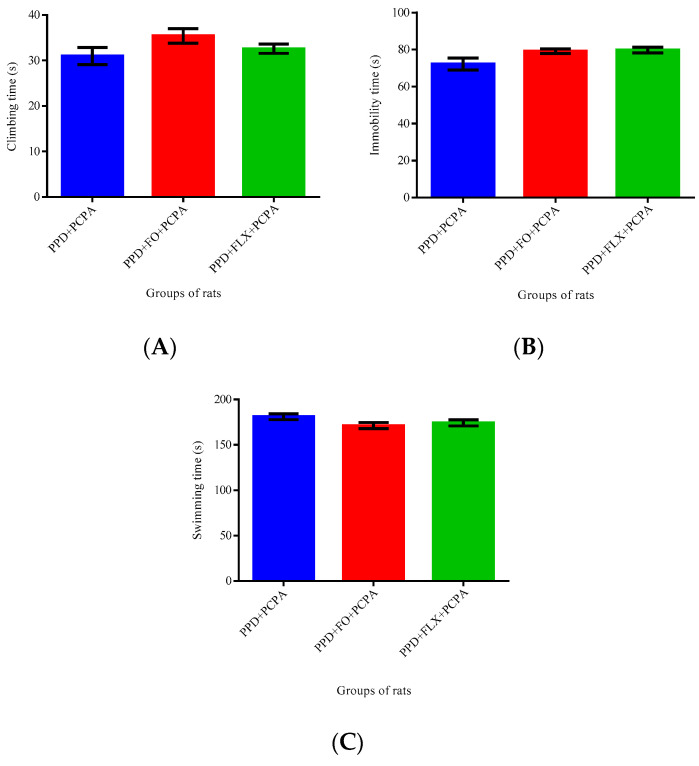
Effect of PCPA on FO and FLX on Forced swimming test in PPD-like rats. (**A**), climbing time. (**B**), Immobility time and (**C**), Swimming time. One-way ANOVA revealed no statistically significant differences among the rat groups. Data presented as mean ±SEM, *n* = 6. (PPD—postpartum depression-like, FO—fish oil, FLX—fluoxetine, PCPA—p-cholorophenylalanine).

Figure 4 have shown the effects of 3 days administration of PCPA on behaviors of FO and FLX administered PPD rats. PCPA, being a serotonin blocker, blocked the effects of both FO and FLX on the climbing [F (2, 12) = 2.09, *p* = 0.166], immobility [F (2, 12) = 3.63, *p* = 0.06] and swimming [F (2, 12) = 2.27, *p* = 0.144] behaviors of the rats, for there was no statistically significant difference among them revealed by one-way ANOVA.

#### 3.1.3. Open Field Test

**Figure 5:**
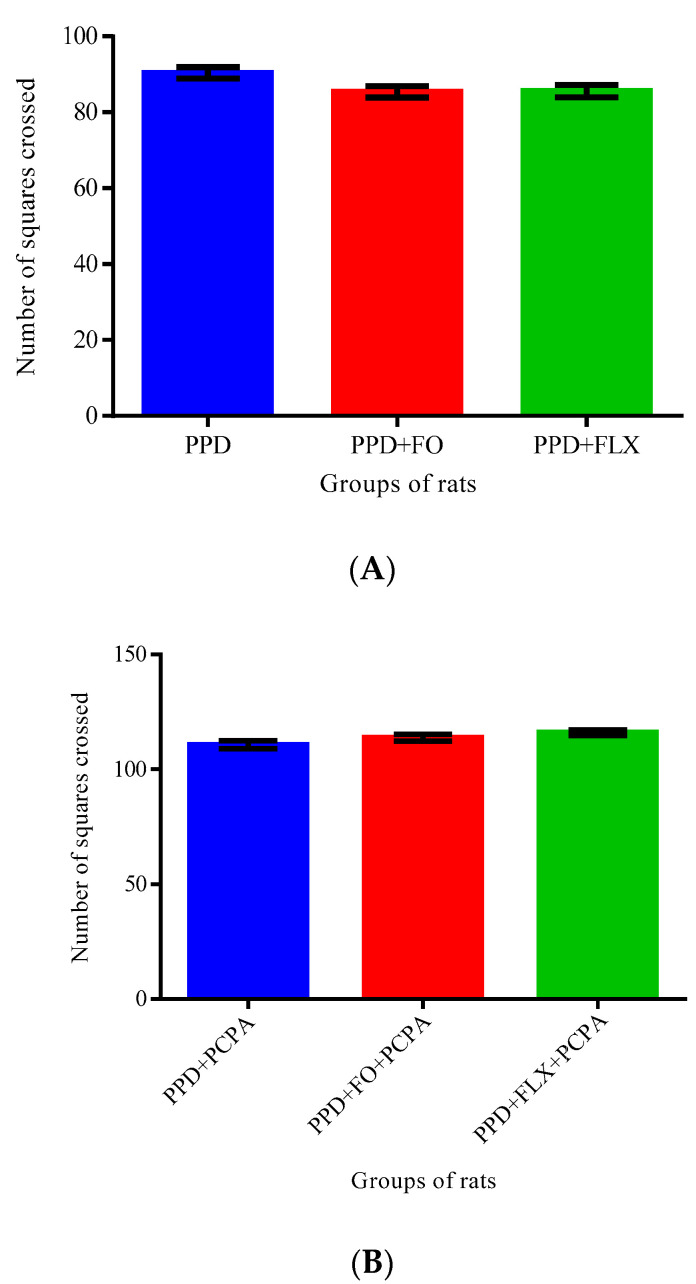
Effect of PCPA on FO and FLX on open field test (OFT) in PPD-like rats. (**A**)—Protocol 1. (**B**)—Protocol 2. (PPD—postpartum depression-like, FO—fish oil, FLX—fluoxetine, PCPA—p-cholorophenylalanine).

Figure 5A,B showed the number of squares crossed by PPD-like rats supplemented by FO and FLX and those PPD-like rats supplemented with FO and FLX but also administered with PCPA for 3 days. One-way ANOVA did not reveal any statistically significant differences in the number of squares crossed by all the rat groups in both the first [F (2, 12) = 3.348, *p* = 0.06] and second [F (2, 12) = 3.060, *p* = 0.084] protocols.

### 3.2. Effects of 10 Days Supplementation of FO on PPD-Like Rats’ Level of 5HT, 5HIAA and 5HIAA/5HT Concentration in Their Prefrontal Cortex and Hippocampus

Table 2 showed the concentration of 5HT, 5HIAA and 5HIAA/5HT in PPD-like groups of rats in protocol 1 and protocol 2. One-way ANOVA showed no statistically significant changes in the concentration of 5HT, 5HIAA and 5HIAA/5HT among all the groups of rats in both protocols in their prefrontal cortex [F (5, 30) = 0.820, *p* = 0.545]. However, one-way ANOVA revealed statistically significant changes in the concentrations of 5HT, 5HIAA and 5HIAA/5HT in their hippocampus. A significant reduction (*p* < 0.05) in the concentration of 5HIAA was observed in the hippocampus of the PPD-like + FO and PPD-like + FLX groups of rats when, compared to the PPD-like model group of rats, as revealed by Tukey’s post hoc test. Treatment with PCPA demolished the effect of FO and FLX on the rats, as no marked changes in the concentration of 5HIAA were observed in the hippocampus of PPD-like + PCPA, PPD-like + FO + PCPA or PPD-like + FLX + PCPA groups of rats. Further, a significant reduction (*p* < 0.05) in serotonin turnover was observed in the hippocampus of the PPD-like + FO and PPD-like + FLX groups of rats, when compared to the PPD-like group of rats. Nevertheless, treatment with PCPA demolished the effects of FO and FLX, as there was no significant difference observed in serotonin turnover among the PPD-like + PCPA, PPD-like + FO + PCPA and PPD-like + FLX + PCPA groups of rats.

## 4. Discussion

The findings from this study showed that the antidepressant-like effects of FO after 10 days of supplementation to PPD-like rats was influenced by its ability to regulate the level of serotonin in their brain, as treating the PPD-like rats with a PCPA inhibitor, tryptophan hydroxylase, an enzyme responsible for producing serotonin, successfully reversed the effects of the FO on the PPD-like rats.

FST is one of the widely accepted methods for the assessment of depressive-like behaviors and for the screening of antidepressants in rodents [26,27]. It is based on the principles that animals, in an enclosed environment, become immobile after an initial period of strenuous activity and the duration of immobility decreases with the administration of effective antidepressants [23]. The FST conducted in this study showed that supplementation with FO reduces the immobility time and increases the swimming time of the PPD-like rats, also decreasing their climbing time. The authors were surprised to find the decrease in climbing time in the rats administered with FO, when compared to the PPD-like model group. However, Cryan [28] reported that noradrenergic compounds selectively increase climbing behavior, compared to the increase in swimming behavior observed in serotonergic compounds. The shorter climbing time observed in the present study suggests that the intervention administered might not be working through the noradrenergic pathway. Therefore, the FO administered in the present study could be working through the serotonergic system, since it increased swimming time. This assertion conforms with Azmitia [29] and Detke [18] who reported that serotonergic antidepressants, including SSRIs, selectively increase swimming behavior in rats. Under the experimental conditions of this study, the induction of PPD and administration of FO or FLX or induction of PPD with administration of FO + PCPA or FLX + PCPA did not interfere with the locomotor activities of the rats, as shown in Figure 5. Therefore, impairments of motor activity are not likely to affect the depression test carried out. Similar outcomes have been documented in other behavioral studies [30,31].

The antidepressant potentials of FO have been highlighted by previous studies [11,16,32]. Additionally, 15 days of supplementation of FO as a source of omega-3 fatty acid to PPD-like rats have successfully reduced their immobility time in FST [13,17]. However, there is paucity of information with regards to the effects of FO on active behaviors, swimming and climbing time in PPD-like rats, as increased swimming time and decreased immobility time in FST is an indicator that the rats being tested are less depressed [33]. The present study showed that FO significantly increased the swimming time, but decreased the climbing time of the PPD-like rats. This suggests that the 10 days of supplementation of FO to the PPD-like rats is effective, as comparable to fluoxetine, an antidepressant drug from the selective serotonin reuptake inhibitors group.

There are usually distinct patterns of active behaviors, either swimming or climbing, in FST as a result of the type of antidepressant being used. Predominantly, in FST, antidepressants that increase serotonergic neurotransmitter show increased swimming time, whereas the antidepressants that increases catecholaminergic neurotransmission increase the climbing time of the PPD-like rats [28]. Therefore, this study suggested that FO antidepressant-like effects were influenced by serotonergic system; thus, inhibition of serotonin production will interfere with efficacy of FO as antidepressant agent. Similar studies also showed that the antidepressant-like effects of FO were associated with increased swimming time in FST and were influenced by serotonergic neurotransmitter [16]. The association of the modulation of serotonin on depression and PPD has not been reported by many investigators; further studies are recommended in order to ascertain a clearer picture of the interaction between serotonin concentration and PPD [34].

Serotonin is a chemical messenger responsible for neuronal function and cell signaling [35]. It is one of the most studied neurotransmitters and has been extensively used in antidepressant drugs for treating depression as SSRIs. Antidepressant drugs applied the concept of increasing serotonin concentration at the synaptic cleft of a neuron to exert their effects. In depressed animals, the level of monoamines neurotransmitters will be decreased and therefore most of the current antidepressant drugs used apply the concept of alteration of monoamine neurotransmitter concentration levels [36]. Low level of serotonin has been found to increase the risk of depression in susceptible postpartum women [37]. In postpartum depressed rats, the concentration of serotonin was suppressed [38]. This was supported by a case-control study in China that mentioned lower serotonin concentration was linked to PPD development [39]. In line with the behavioral results of this study, neurochemicals observation showed that FO significantly decreased the turnover rate of serotonin in the PPD-like rat’s hippocampus, as an increased turnover rate of serotonin has been shown to be associated with high risk of depression [40]. However, by administering PCPA to PPD rats in this study, the antidepressant-like effects of FO were reversed, as reduction of serotonin was followed by higher immobility time in FST, which indicated higher depressive-like behavior [41]. In PPD experimental subjects who had involved the hormones fluctuation approach, the concentration of serotonin was affected greatly [42]. In PPD, low concentration of serotonin at the early weeks of postpartum has been suggested to influence its onset [43].

FO is a potential supplement in managing depression development [44] and the administration of FO has been widely related to its benefits as an antidepressant agent. The current study brought to the limelight the association of FO antidepressant-like effects in PPD-like rats involving serotonergic monoamine serotonin.

## 5. Conclusions

As a conclusion, this study showed that 10 days of supplementation of FO to PPD-like rats confers antidepressant-like effects on them, which are comparable to that of FLX, as confirmed by the decreased immobility and increased swimming times in FST. This was achieved through the modulation of serotonin neurotransmitters in the hippocampus of the PPD-like rats. Therefore, the results suggested the involvement of the serotonergic system in the FO antidepressant-like effects on the PPD-like rats. However, this current study is limited to the serotonergic pathway; therefore, involvement of other pathways, such as neuroinflammation on the effects of FO on PPD-like rats, should be explored in future studies.

## Figures and Tables

**Table 1:** Composition of rat chow (Gold coin Malaysia) used for the feeding of the rats [21].

Composition	Amount/Percentage
Crude protein	21–23%
Crude fiber (max)	5.0%
Crude fat (min)	3.0%
Moisture (max)	3.0%
Calcium	0.8–1.2%
Phosphorus	0.6–1.0%
Nitrogen free extract	49.0%
Vitamin A	10 M.I.U.
Vitamin D_3_	2.5 M.I.U
Vitamin E	15 g
Vitamin K	trace
Vitamin B_12_	trace
Thiamine	trace
Riboflavin	trace
Pantothenic acid	trace
Niacin	trace
Pyridoxine	trace
Choline	trace
Santoquin	trace
Microminerals	trace

**Table 2:** Biochemical analysis of 5HT, 5-HIAA concentration (ng/mL) and 5HIAA/5HT on PFC and hippocampus of PPD-like rats supplemented with FO. Tukey’s post hoc was used for the comparisons.

Groups	PPD	PPD + FO	PPD + FLX	PPD + PCPA	PPD + FO + PCPA	PPD + FLX + PCPA
**Prefrontal Cortex**						
**5HT**	45.03 ± 2.1	48.60 ± 3.6	48.40 ± 4.7	47.98 ± 5.3	40.02 ± 2.1	42.42 ± 4.6
**5HIAA**	13.18 ± 1.2	12.63 ± 1.9	10.68 ± 0.7	10.92 ± 0.9	10.60 ± 1.1	10.35 ± 1.0
**5HIAA/5HT**	29.78 ± 3.3	27.30 ± 5.2	23.31 ± 2.9	15.53 ± 1.9	26.85 ± 3.1	25.77 ± 3.4
**Hippocampus**						
**5HT**	42.80 ± 2.1	49.98 ± 3.4	49.48 ± 4.0	44.25 ± 2.4	43.77 ± 4.5	44.7 ± 0.8
**5HIAA**	12.67 ± 1.9	6.02 ± 0.3 ^#^	5.78 ± 0.6 ^#^	10.97 ± 1.1	9.85 ± 1.1	11.067 ± 0.7
**5HIAA/5HT**	19.57 ± 2.9	12.7 ± 1.1 ^#^	11.7 ± 0.9 ^#^	26.24 ± 2.7	25.26 ± 5.5	26.23 ± 1.9

(^#^) significant difference at *p* < 0.05 compare to PPD group.
